# Real impact of liver cirrhosis on the development of hepatocellular carcinoma in various liver diseases—meta‐analytic assessment

**DOI:** 10.1002/cam4.1998

**Published:** 2019-02-21

**Authors:** Kazuo Tarao, Akito Nozaki, Takaaki Ikeda, Akira Sato, Hirokazu Komatsu, Tatsuji Komatsu, Masataka Taguri, Katsuaki Tanaka

**Affiliations:** ^1^ Tarao’s Gastroenterological Clinic Yokohama Japan; ^2^ Gastroenterological Center, Medical Center Yokohama City University Yokohama Japan; ^3^ Gastroenterology Department Yokosuka General Hospital Uwamachi Yokosuka Japan; ^4^ Division of Gastroenterology, Department of Internal Medicine St. Marianna University, Yokohama City Seibu Hospital Yokohama Japan; ^5^ Department of Gastroenterology Yokohama Municipal Citizen’s Hospital Yokohama Japan; ^6^ Department Clinical Research National Hospital Organization, Yokohama Medical Center Yokohama Japan; ^7^ Department of Data Science Yokohama City University Yokohama Japan; ^8^ Hatano Red Cross Hospital Kanagawa Japan

**Keywords:** hepatocellular carcinoma, liver cirrhosis, liver diseases, meta‐analysis, risk of HCC

## Abstract

**Background:**

It is well known that the incidence of developing hepatocelluler carcinoma (HCC) is increased in liver cirrhosis of different etiologies. However, comparison of HCC incidence in various liver diseases has not yet been estimated. We surveyed this comparison.

**Methods:**

The PubMed database was examined (1989‐2017) for studies published in English language regarding the prospective follow‐up results for the development of HCC in various liver diseases. A meta‐analysis was performed for each liver disease.

**Results:**

The annual incidence (%) of HCC in the non‐cirrhotic stage and cirrhotic stage, and the ratio of HCC incidence in the cirrhotic stage/non‐cirrhotic stage were as follows. (a) hepatitis B virus liver disease: 0.37%→3.23% (8.73‐fold), (b) hepatitis C virus liver diseases: 0.68%→4.81% (7.07‐fold), (c) primary biliary cholangitis (0.26%→1.79%, 6.88‐fold), (d) autoimmune hepatitis (0.19%→0.53%, 2.79‐fold), and (e) NASH (0.03%→1.35%, 45.00‐fold). Regarding primary hemochromatosis and alcoholic liver diseases, only follow‐up studies in the cirrhotic stage were presented, 1.20% and 2.06%, respectively.

**Conclusions:**

When the liver diseases advance to cirrhosis, the incidence of HCC is markedly increased. The development of HCC must be closely monitored by ultrasonography, magnetic resonance imaging, and computed tomography, irrespective of the different kinds of liver diseases.

## 1 INTRODUCTION

It is well known that cirrhosis is the most potent risk factor for the development of hepatocellular carcinoma (HCC), irrespective of the etiology of liver disease. However, precise comparison of the incidence of HCC in various liver diseases especially in liver cirrhosis (LC) has not yet been elucidated. Moreover, the degree of increase in cirrhotic state in various liver diseases has not also yet been estimated. In this study, we compared the incidence of HCC in LC in various liver diseases by meta‐analysis, and also surveyed how the incidence of developing HCC is increasing in the cirrhotic state as compared with that in the non‐cirrhotic state in various liver diseases. Furthermore, we discuss the possible mechanisms of HCC development in various liver diseases.

## 2 MATERIAL AND METHOD

### 2.1 Search strategy

The PubMed database was searched (1989‐2017) for studies published in English regarding the follow‐up results for the development of HCC in various liver diseases to perform meta‐analyses. Only prospective studies for the development of HCC were used, and retrospective studies were omitted. In this search, review articles were omitted. Among studies on hepatitis B virus (HBV) and hepatitis C virus (HCV) infections, those associated with therapeutic intervention were excluded, and studies that followed the natural course were included. Among reports on primary biliary cholangitis, the major pathological classification was Sheuer's Ⅰ/Ⅱ and Ⅲ (pre‐cirrhosis)/Ⅳ (cirrhosis) in almost all papers demonstrated. Thus, the ratio of HCC incidence represents Sheuer's Ⅲ/Ⅳ compared with Ⅰ/Ⅱ.

### 2.2 Statistical analysis

For the seven diseases, we calculated the weighted mean of the HCC incidence rate for LC and non‐LC using the random effects model (ref: Dersimonian R, Laird N. Meta‐analysis in clinical trials. Controlled Clinical Trials. 1986; 7:177‐188). To assess whether the incidence rate among LC patients was higher than that among non‐LC patients, we calculated the incidence rate ratio and *P*‐value using the *Z*‐test. All reported *P*‐values correspond to two‐sided tests, and those <0.05 were considered significant. Analyses were performed with JMP version 12 (SAS Institute, Cary, NC).

## 3 RESULTS

### 3.1 Incidence of HCC according to the etiology of cirrhosis

#### 3.1.1 Incidence of HCC in HBV infection

**Figure 1:**
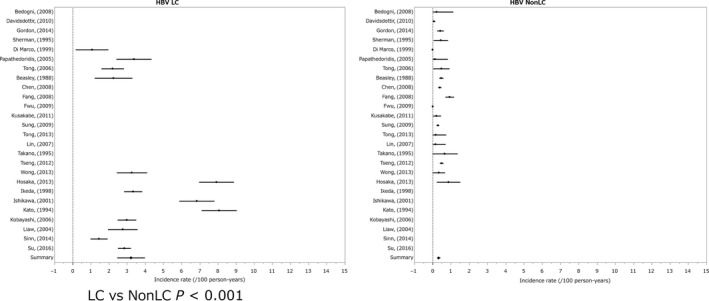
Incidence of hepatocelluler carcinoma in hepatitis B virus infection

The annual incidence (%) of HCC in the non‐cirrhotic state was 0.37% and that in the cirrhotic state was 3.23% (LC vs non‐LC *P* < 0.001).^1^, ^2^, ^3^, ^4^, ^5^, ^6^, ^7^, ^8^, ^9^, ^10^, ^11^, ^12^, ^13^, ^14^, ^15^, ^16^, ^17^, ^18^, ^19^, ^20^, ^21^, ^22^, ^23^, ^24^, ^25^ The ratio of HCC incidence for the cirrhotic state/non‐cirrhotic state was 8.73‐fold. In this group, cases with interventional therapy were excluded. The majority of reports describing the follow‐up results of non‐cirrhotic HB patients included follow‐up results of patients with chronic hepatitis B, and not inactive HBV healthy carriers (Figure 1).

#### 3.1.2 Incidence of HCC in HCV infection

**Figure 2:**
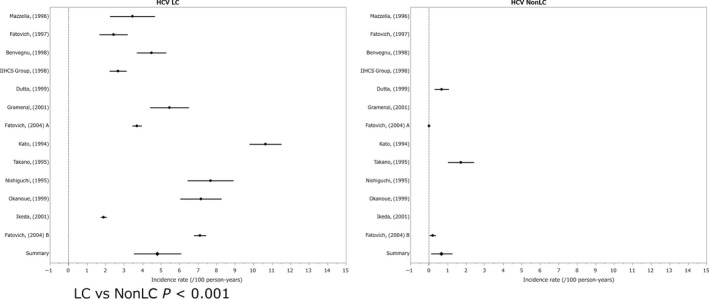
Incidence of hepatocelluler carcinoma in hepatitis C virus infection

The annual incidence (%) of HCC in the non‐cirrhotic state was 0.68% and that in the cirrhotic state was 4.81% (LC vs non‐LC *P* < 0.001).^26^, ^27^, ^28^, ^29^, ^30^, ^31^, ^32^, ^33^, ^34^, ^35^, ^36^, ^37^, ^38^, ^39^, ^40^, ^41^, ^42^, ^43^, ^44^, ^45^, ^46^, ^47^, ^48^, ^49^, ^50^, ^51^, ^52^, ^53^ The ratio of HCC incidence for cirrhotic state/non‐cirrhotic state was 7.07‐fold. In this group, patients with interventional therapy were excluded. The majority of the non‐cirrhotic patients had chronic hepatitis C (Figure 2).

#### 3.1.3 Incidence of HCC in primary biliary cholangitis (PBC)

**Figure 3:**
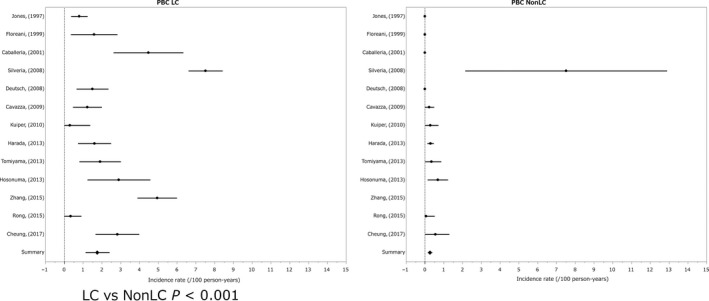
Incidence of hepatocelluler carcinoma in primary biliary cholangitis

The annual incidence (%) of HCC in the non‐cirrhotic state (Scheuer's stage Ⅰ~Ⅱ) was 0.26% and that in the pre‐cirrhotic ~cirrhotic state (Scheuer's stage Ⅲ~Ⅳ) was 1.79% (LC vs non‐LC *P* < 0.001).^54^, ^55^, ^56^, ^57^, ^58^, ^59^, ^60^, ^61^, ^62^, ^63^, ^64^, ^65^, ^66^, ^67^, ^68^, ^69^, ^70^, ^71^, ^72^, ^73^, ^74^ The ratio of HCC incidence for stage Ⅲ~Ⅳ/stageⅠ~Ⅱ was 6.88‐fold. In the majority of patients with PBC, the background of the liver was in cirrhotic state when HCC developed. The majority of patients with PBC was treated with ursodeoxycholic acid in both cirrhotic and non‐cirrhotic patients (Figure 3).

#### 3.1.4 Incidence of HCC in autoimmune hepatitis

**Figure 4:**
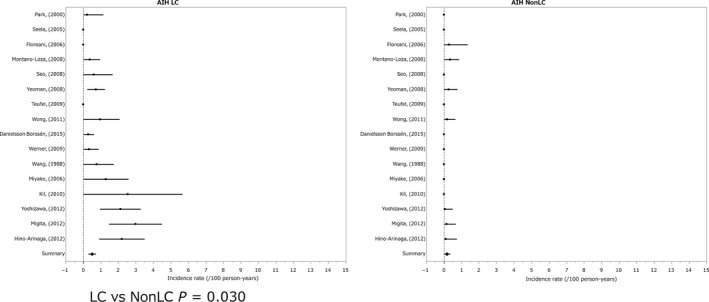
Incidence of hepatocelluler carcinoma in autoimmune hepatitis

The annual incidence (%) of HCC in the non‐cirrhotic state was 0.19% and that in the cirrhotic state was 0.53% (LC vs non‐LC *P* = 0.030).^75^, ^76^, ^77^, ^78^, ^79^, ^80^, ^81^, ^82^, ^83^, ^84^, ^85^, ^86^, ^87^, ^88^, ^89^, ^90^ The ratio of HCC incidence for the cirrhotic state/non‐cirrhotic state was 2.79‐fold (Figure 4).

#### 3.1.5 Incidence of HCC in nonalcoholic steatohepatitis (NASH)

**Figure 5:**
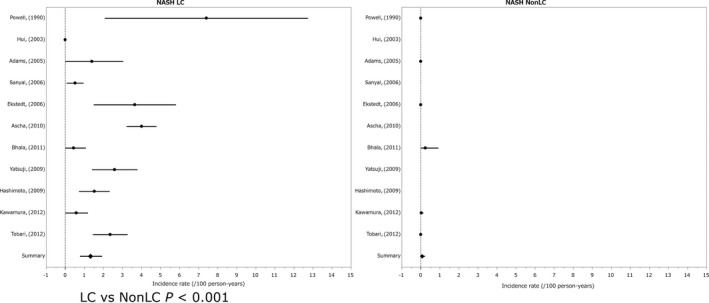
Incidence of hepatocelluler carcinoma in nonalcoholic steatohepatitis

The annual incidence (%) of HCC in the non‐cirrhotic state was 0.03% and that in the cirrhotic state was 1.35% (LC vs non‐LC *P* < 0.001).^91^, ^92^, ^93^, ^94^, ^95^, ^96^, ^97^, ^98^, ^99^, ^100^, ^101^ The ratio of HCC incidence for the cirrhotic state/non‐cirrhotic state was 45.00‐fold. Of the NASH patients, 52% had diabetes mellitus and 81% were obese (Figure 5).

#### 3.1.6 Incidence of HCC in genetic hemochromatosis

**Figure 6:**
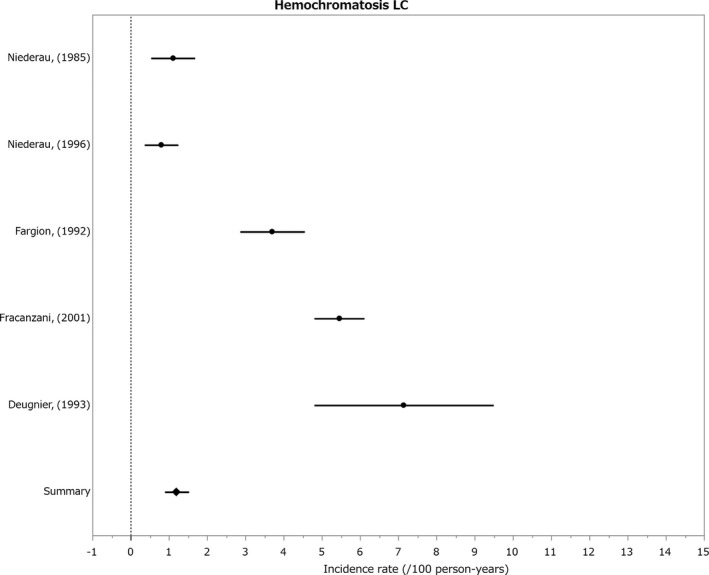
Incidence of hepatocelluler carcinoma in primary hemochromatosis

Only follow‐up studies for the cirrhotic state were reported and the incidence was 1.20%/year (Figure 6).^102^, ^103^, ^104^, ^105^, ^106^, ^107^, ^108^, ^109^

#### 3.1.7 Incidence of HCC in alcoholic liver disease

**Figure 7:**
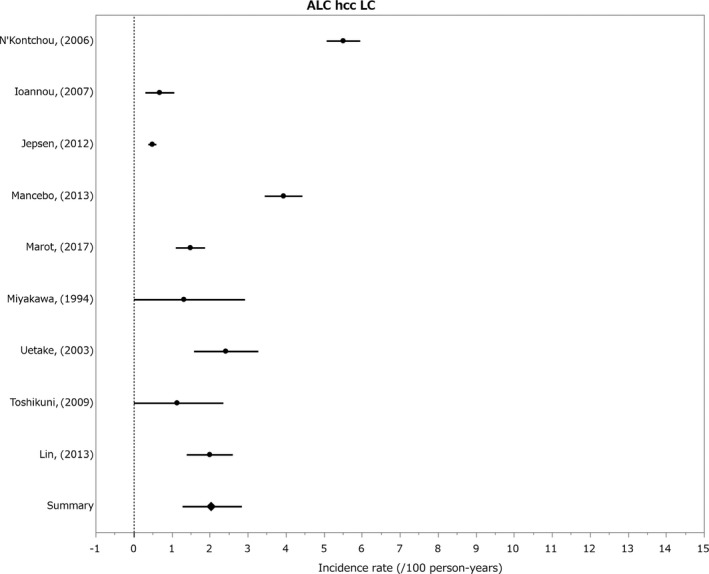
Incidence of hepatocelluler carcinoma in alcoholic liver diseases

Only follow‐up studies for alcohol‐related cirrhosis were reported and the incidence was 2.06% (Figure 7).^110^, ^111^, ^112^, ^113^, ^114^, ^115^, ^116^, ^117^, ^118^

The above results are summarized in the Table 1.

**Table 1:** Incidence of HCC (%/year) and rate ratio (LC/non LC) in liver diseases

	Incidence rate (%/year)		Rate/ratio (LC/non LC)
	LC	Non LC	
HBV infection	3.23	0.37	8.73
HCV infection	4.81	0.68	7.07
PBC	1.79	0.26	6.88
AIH	0.53	0.19	2.79
NASH	1.35	0.03	45.00
Primary hemochoromatosis	1.20	—	—
Alcholic liver diseases	2.06	—	—

HCC, hepatocelluler carcinoma; LC, liver cirrhosis.

## 4 DISCUSSION

It is known that cirrhosis is present in 80～90% of HCC patients with any underlying liver disease,^119^ and it is the most important risk factor for HCC. However, comparison of the incidence of HCC in various liver diseases was not accurately and precisely evaluated in previous studies. In this study, we found that the incidence of HCC is highest in HCV LC (4.81%/year) and second highest in HBV LC (3.23%), followed by alcoholic LC (2.06%), PBC LC (1.79%), NASH LC (1.35%), primary hemochromatosis (1.20%), and AIH (0.53%).

The incidence of HCC has been wildly studied in patients with HBV LC and HCV LC, and was reported to be 3% and 5%,^29^, ^120^ which was almost the same as that in our study.

In this study, we also demonstrated that the incidence of HCC is markedly increased (2.79‐ to 45.00‐fold) in the cirrhotic state compared with non‐cirrhotic state, irrespective of the etiology of liver disease. Why this increase in HCC development occurs in the cirrhotic state must be considered.

First, chronic inflammation may be a key mechanism for HCC development in the cirrhotic state. In this respect, we made clinical observation in the HCV LC patients (Child A) in the past. The LC patients were divided into three groups: Group A: 28 patients whose annual average serum alanine aminotransferase (ALT) level was persistently high (≧80 IU; high‐ALT group); Group B: 28 patients whose annual average serum ALT levels was persistently low (<80 IU; low‐ALT group), and Group C: 13 unclassified patients. The patients had been followed up prospectively. The 5‐year incidence rate of HCC in the high‐ALT group was as high as 53.6% compared with only 7.1% in the low‐ALT group (*P* < 0.001).^120^ Thus, this clinical observation demonstrated the importance of chronic inflammation in the development of HCC in HCV LC.

The same tendency was demonstrated in HBV LC. Chen et al^121^ reported that high serum levels of HBV DNA and ALT were independent risk factors for HCC development in HBV infection. Ishikawa et al^20^ also reported that serum aminotransferase are one of predictive factor for the development of HCC. One important mechanism for high incidence of HCC in HCV LC and HBV LC as compared with the incidence of HCC in LC caused by other etiologies may be the sustained high‐grade inflammation.

Furthermore, in autoimmune hepatitis, persistent elevation of serum aminotransaminase was reported to lead to the development of HCC.^78^ This suggests a role for inflammation in the development of HCC, but this hypothesis has not been confirmed solidly.

Second, increases in DNA synthesis in the hepatocytes of cirrhotic patients is suspected as a possible mechanism of HCC development. In our previous study, we demonstrated that in the high DNA synthetic group [BrdU (thymidine analog) labeling index ≧1.5%] 64.3% of patients developed HCC in 5 years, in contrast to 14.3% in the low DNA synthesis (Brd U LI <1.5%) group in HCV LC patients (*P* < 0.05).^122^ We further demonstrated that in HCV‐associated cirrhosis patients who showed nodular formation on ultrasound, the cell cycle of hepatocytes was markedly accelerated, as estimated by the bromodeoxyuridine (thymidine analog) uptake into hepatocytes in vitro, and the incidence of HCC was greatly increased.^123^

Third, accumulated genomic change was also important factor for HCC development. In this respect, Hiatt et al^124^ reported that C282Y mutation itself may increase the risk of HCC development in hereditary hemochromatosis. In addition, in alcoholic LC, the genetic effect of long‐term alcoholic intake may influence the development of HCC.^125^

Fourth, obesity, and diabetes are suspected to increase the incidence of developing HCC. In a large epidemiologic study, patients with BMI >35 had an increased risk of cancer, especially HCC, with hazard ratio (HR) of 4.52.^126^ In addition, diabetes is associated with HCC occurrence, with a HR of 2.39 in a US cohort.^127^ It is generally accepted that NASH is associated with obesity and diabetes in high percentages.

Notably, in 2014, Flemming et al^128^ predicted the risk of HCC in patients with cirrhosis, using the ADRESS‐HCC risk model. They examined the l‐year probability of HCC in various liver diseases in 34 932 patients in the national transplantation waitlist database. HCC developed in 1960 patients (5.6%) during a median follow‐up of 1.3 years. Six baseline clinical variables including age, diabetes, race, etiology of cirrhosis, sex, and severity (ADRESS) of liver dysfunction were independently associated with HCC. They settled ADRESS‐HCC risk model from these data and the risk model well‐coincided with the clinical observation. They concluded that the risk model was clinically useful tool for predicting the risk of HCC in patients with diverse etiologies.

In addition, the aging of the patients must be taken into the consideration, as the cirrhotic patients were considered to be older than the non‐cirrhotic patients in almost all liver diseases. In this regard, Asahina et al^129^ investigated the difference of HCC incidence in aging in HCV‐associated liver disease, and found that the incidence of HCC was higher in the older patients (>65 years old) than the younger patients (<65 years old). The same tendency was observed by Taura et al^130^ However, the difference in incidence was approximately twofold. So, it is difficult to explain the marked difference in HCC incidence between the cirrhotic state and non‐cirrhotic state found in this meta‐analysis. Regarding other liver diseases, there were very few reports which demonstrated a difference between patients in the non‐cirrhotic and cirrhotic states concerning age.

The next consideration is the prevention of patients not to become cirrhosis state. We demonstrated that the incidence of HCC in the cirrhotic state and that in the non‐cirrhotic state were markedly different in many liver diseases; therefore, efforts to prevent patients with liver diseases from progressing to the cirrhotic state by all means is very important.

In HBV infection, the effort to diminish HBV‐DNA by pegylated interferon (Peg IFN)^19^ or oral antiviral agents, such as entecavir (ETV), tenofovir, and tenofovir anafenamide, is important. Indeed, ETV and tenofovir were reported to prevent the occurrence of HCC.^131^, ^132^

In HCV infection, Peg IFN plus ribavirin or direct‐acting antivirals (DAAs) are effective to eliminate HCV, and may be prevent the progression of the disease, resulting in prevention of HCC development.

In primary biliary cholangitis, administration of ursodeoxycholic acid is important. Indeed, the risk of HCC in UDCA‐treated PBC patients was reported to be relatively low.^59^, ^63^

In autoimmune hepatitis, well‐designed corticosteroid therapy is important to prevent HCC development, and persistent elevation of serum aminotransaminase is reported to lead the development of HCC.^78^ It seems that alleviation of serum ALT levels might prevent HCC development, but this hypothesis is not confirmed solidly.

In NASH patients, improvement in metabolic syndrome, especially improvement in diabetes mellitus and obesity, is important.

Next, we mentioned whether the treatment of underlying liver diseases substantially modulates the HCC risk or not. We restricted the survey chiefly to the cirrhotic patients because they are at high risk of HCC development.

### 4.1 HBV‐related cirrhosis

#### 4.1.1 Interferon (IFN)

Ikeda et al^19^ investigated influence of long‐term IFN administration on the rate of occurrence of HCC in patients with HBV‐related cirrhosis and found that cumulative occurrence rates of HCC in the group treated with IFN and the untreated group were 4.5% and 13.3%, respectively, at the end of 3 years; 7.0% and 19.6%, respectively, at the end of 5 years; and 17.0% and 30.8%, respectively, at the end of 10 years. The rate of HCC development in the treated group was significantly lower than that of the untreated group (*P* = 0.0124). Interferon treatment was an independent contributing factor in lowering the rate of carcinogenesis (odds ratio = 0.39; *P* = 0.031). The same tendency was observed by Lin et al^14^

#### 4.1.2 Oral nucleos(t)ide analogues

Wong et al^17^ compared the incidence of HCC between the 482 ETV‐treated HBV cirrhotic patients and 69 untreated patients, and found that the 5‐year cumulative incidence of HCC was 13.8% in the ETV‐treated and 26.4% in the untreated patients (*P* = 0.036) and concluded that ETV treatment reduces HCC development in chronic hepatitis B patients with liver cirrhosis. The same tendency was observed by Su et al^25^

Furthermore, Hosaka et al^18^ compared the incidence of HCC between lamivudine (LAM)‐treated, ETV‐treated HBV‐cirrhotic patients, and control HBV cirrhotic patients, and found that the cumulative incidence of HCC in 5 years were 22.2% in the LAM‐treated, 7% in the ETV‐treated, and 38.9% in the control groups, and HCC suppression effect was greater in the ETV‐treated group (*P* < 0.001) than in the LAM‐treated group (*P* = 0.019) and the control group.

Additionally, Kim et al^131^ examined the impact of tenofovir disoproxil fumarate (TDF) on the incidence of HCC, and found that the long‐term (384 weeks) therapy with TDF was associated with the reduced incidence of HCC among patients without cirrhosis.

### 4.2 HCV‐related cirrhosis

#### 4.2.1 Interferon

Nishiguchi et al^27^ investigated the effects of IFN‐α on the development of HCC in the HCV‐related cirrhotic patients. IFN‐α (6 MU three times weekly) was administered for 12‐24 weeks in 45 Child A cirrhotic patients and compared with the 45 patients with symptomatic treatment. HCC was detected in 4% of IFN patients and 38% in controls (*P* = 0.002), and the risk ratio of IFN‐α treatment vs symptomatic treatment was 0.067.

Ikeda et al^47^ also investigated the effect of IFN on HCC development in the HCV‐related cirrhosis. HCC developing rates in the untreated and IFN groups were 14.8 and 9.1% at the end of 3rd year, 28.4 and 14.1% at the end of the 5th year, and 52.5 and 36.7% at the 10th year, respectively. The carcinogenesis rate of the IFN‐treated group was significantly lower than that of the untreated group (*P* = 0.0028). The same tendency was observed by Benvegnú et al^33^ and Yoshida et al^42^

#### 4.2.2 Direct‐acting antiviral agents

Kanwal et al^133^ compared the risk of HCC in patients with vs those without sofosbuvir (SVR) (chiefly sofosbuvir + ledipasvir).

Nineteen thousand five hundred and eighteen patients with sustained virological response (SVR) and 2,982 patients without SVR (39.0% had cirrhosis) were included. Compared with patients without SVR, those with SVR had a significantly reduced risk of HCC (0.90 vs 3.45 HCC/100 person‐years, HR 0.28). Moreover, the magnitude of SVR protective effect was similar in patients with and without cirrhosis (HR = 0.32 and HR = 0.18).

Furthermore, Ioannou et al^134^ investigated the impact of DAA‐induced SVR on HCC risk. They included 35,871 (58%) IFN (chiefly Peg IFN)‐only regimens, 4,535 (7.2%) DAA + IFN regimens, and 21,948 (35%) DAA (chiefly sofosbuvir/ledipasvir)‐only regimens. They retrospectively followed up patients for a mean follow‐up period of 6.1 years. SVR was associated with a significantly decreased risk of HCC irrespective of whether antiviral treatment was DAA‐only (adjusted hazard ratio [AHR] 0.29), DAA + IFN (AHR 0.48), or IFN‐only (AHR 0.32). DAA‐induced SVR is associated with a 71% reduction in HCC risk. Treatment with DAAs is not associated with increased HCC risk compared with treatment with IFN. Patients who achieved SVR had a lower incidence of HCC compared with those who did not achieve SVR among both patients with cirrhosis and those without.

### 4.3 Primary biliary cirrhosis

Jackson^58^ investigated whether the risk of developing HCC is reduced by use of ursodeoxycholic acid. They categorized the patients into regular ursodeoxycholic acid–treated patients (with six or more prescriptions) and nonregular‐treated patients (with less than six), and compared the incidence of HCC. The increased risk of HCC in regular ursodeoxycholic acid–treated patients as compared with control subjects was threefold (HR, 3.17) in contrast to an eightfold increase (HR, 7.77) in those nonregular treated.

Furthermore, Kuiper et al^62^ investigated the risk factor for HCC development in PBC patients, and found that the strongest risk factor for HCC was the absence of biochemical response after 1‐year treatment with UDCA (*P* < 0.001). (The response to UDCA was defined as normalization of bilirubin and/or albumin levels after administration of UDCA for one year.)

### 4.4 Autoimmune cirrhosis

There are no reports which deals with the incidence of HCC in the untreated autoimmune patients. However, there are some studies which showed increased incidence of HCC in patients who showed no effectiveness to immune suppressive agent and had continued active hepatitis.

Yoshizawa et al^88^ followed up 203 well‐defined AIH patients for a mean follow‐up period of 131 months. All patients were treated with corticosteroid with or without azathioprine. They found that the prognosis of two or more relapses were identified as the only risk factor for development of hepatic malignancy (hazard ratio 9.1, *P* = 0.007). Also, Montano‐Loza et al^78^ demonstrated that worsening laboratory tests during corticosteroid therapy was associated with a higher risk of HCC. Moreover, Miyake^86^ et al demonstrated elevation of serum ALT levels leads to HCC development. Furthermore, Hino‐Arinaga et al^90^ surveyed the risk factors for HCC in autoimmune patients and found that cirrhosis at diagnosis of AIH and abnormal ALT at final observation were independently associated with HCC development.

From the above‐mentioned studies, lowering the serum ALT levels, and preventing relapses seemed to be effective to decrease the risk of developing HCC.

### 4.5 NASH cirrhosis

The standard therapy of NASH cirrhosis has not been established completely. However, there is some possibility that improvement in metabolic syndrome, especially improvement in diabetes mellitus and obesity may diminish the incidence of HCC in NASH cirrhosis.

### 4.6 Hereditary hemochromatosis

Niederau et al^103^ followed up 251 patients with hemochromatosis for up to 33 years (mean, 14.1 years). They demonstrated iron removal by phlebotomy therapy markedly improved survival, including liver cirrhosis. They mentioned that all 21 cases of liver cancer developed in cirrhotic livers, and that the development of liver cancer may depend on the amount and duration of iron overload because patients who died from liver cancer had significantly greater iron stores than patients who died from other cancers. From these statements, iron removal by phlebotomy in cirrhotic patients may supposed to decrease the incidence of HCC, although the beneficial effect of iron removal on HCC development has never been proven by controlled trial.

### 4.7 Alcoholic cirrhosis

The published paper dealing with the effect of alcoholic abstinence or diminished alcohol intake on the ratio of HCC development was not confirmed. However, Chuang et al^135^ surveyed by a meta‐analysis the meta‐relative risk (mRR) and dose‐response trend and found that the dose‐response relation between alcohol and development of HCC; the relative risk (RR) was 1.08 for 12 g/day (~1 drink), 1.54 for 50 g/day, 2.14 for 75 g/day, 3.21 for 100 g/day, and 5.20 for 125 g/day of alcohol consumption. It is presumed from these results that the abstinence or diminished alcohol intake would reduce the risk of HCC development.

Another important aspect is surveillance of HCC by imaging modalities, such as ultrasonography (US), magnetic resonance imaging (MRI), or computed tomography (CT) at appropriate intervals (4~6 months) irrespective of the etiology of liver disease when cirrhosis is noted to detect HCC in the early stage.

We found that the incidence of HCC is markedly increased in the cirrhotic state as compared with in the non‐cirrhotic state irrespective of the etiology of liver disease. Therefore, the patients need to be closely monitored for the development of HCC when their liver diseases progress to the cirrhotic state by US, MRI, or CT at appropriate intervals (every 4 months is ideal) to find HCC in the early stage.

## CONFLICT OF INTEREST

Tanaka K has received research funding from Bristol‐Myers Squibb and Abb Vie. Nozaki A has received research funding from Gilead Sciences. Tarao K, Ikeda T, Sato A, Komatsu H, Komatsu T, and Taguri M declare that they have no conflict of interest.
